# Anatomical features of percutaneously closed patent foramen ovale in patients with cryptogenic stroke

**DOI:** 10.1007/s12471-025-01983-y

**Published:** 2025-09-15

**Authors:** Maikel H. M. Immens, Lars S. Witte, Abdelhak el Bouziani, Anthonie Duijnhouwer, Berto J. Bouma, Jan G. P. Tijssen, Frank-Erik de Leeuw, Rob J. de Winter, Tim J. F. ten Cate

**Affiliations:** 1 Department of Neurology, Radboudumc, Donders Institute for Brain, Cognition and Behaviour, Nijmegen, The Netherlands; 2 Department of Cardiology, Amsterdam UMC, University of Amsterdam, Amsterdam Cardiovascular Sciences, Amsterdam, The Netherlands; 3 Department of Cardiology, Radboudumc, Radboud Institute for research and medical innovation, Nijmegen, The Netherlands

**Keywords:** Patent Foramen Ovale, Stroke, Percutaneous closure, Anatomical features

## Abstract

**Background:**

Patent foramen ovale (PFO) is increasingly recognized as a cause of stroke, with a prevalence of approximately 25% in the general population. Consequently, the likelihood of encountering a ‘bystander PFO’ in young patients who have experienced a stroke seems significant. To aid in identifying patients with a PFO-related cryptogenic stroke, an interdisciplinary Heart-Stroke Team (HST) has been established. This team evaluates patients who have suffered from stroke and were diagnosed with a PFO to assess its potential contribution. Understanding the anatomical features of PFOs associated with stroke is essential for decision-making. This study examines the PFO characteristics of all patients who underwent percutaneous PFO closure for cryptogenic stroke at two congenital heart disease institutions in the Netherlands.

**Methods:**

Data on all patients who underwent PFO closure from 2016 to 2022 were collected. Anatomical characteristics were measured using transesophageal echocardiography and analyzed by two cardiologists.

**Results:**

In total, 223 patients underwent PFO closure. The mean age was 42.8 ± 10.7 years, with 115 (51.6%) being male. Approximately 80% of all patients had at least one risk-enhancing PFO feature (moderate to severe shunt and/or atrial septal aneurysm of > 10 mm).

**Conclusion:**

Although all patients accepted for percutaneous PFO closure were individually assessed by a dedicated HST, 20% had a PFO without risk-enhancing features but were still accepted for closure due to other reasons. This highlights the importance of careful individual assessment of young stroke patients with a PFO. Future studies are needed to identify the characteristics that contribute to stroke in these patients.

## Introduction

The prevalence of patent foramen ovale (PFO) in the general population is approximately 25% [1]. PFO-induced right-to-left shunting (RLS) is recognized as a potential contributing factor to stroke, particularly in younger patients between 18 and 60 years of age [2]. A meta-analysis demonstrated that in younger patients (< 55 years) with cryptogenic stroke, the prevalence of PFO was six times higher than in young stroke patients with alternative etiologies [3]. This suggests a possible association between PFO and stroke [4]. Percutaneous closure of PFO has been shown to reduce the risk of recurrent stroke in selected patients [5–7]. Given the high prevalence of PFO in the general population, the likelihood of encountering a ‘bystander PFO’ in young patients who have experienced a stroke seems realistic [8]. To differentiate between patients with a PFO that is considered stroke-related and those with a bystander PFO, the interdisciplinary Heart-Stroke Team (HST) has been established [9]. This team systematically evaluates all stroke patients diagnosed with a PFO to assess its potential contribution to the cerebrovascular event. The evaluation process involves a comprehensive assessment, including neuroimaging, blood tests to screen for coagulation disorders, and a cardiac evaluation to identify potential cardiac sources of stroke. As part of this assessment, echocardiography is performed to confirm the presence of a PFO.

Several anatomical PFO characteristics have been associated with an increased risk of stroke [10]. These include the size of the right-to-left shunt (RLS) and various other anatomical characteristics [11–14], which collectively enhance the likelihood that the PFO is causally related to stroke [10].

**Fig. 1:**
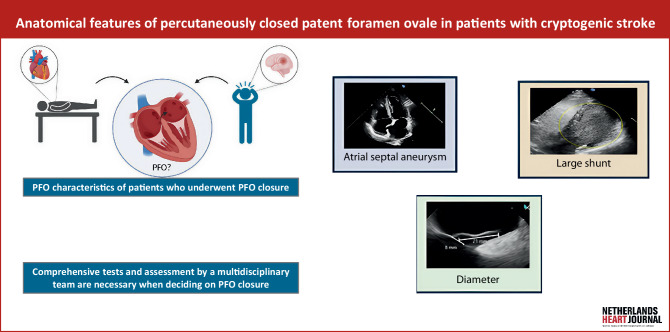
Infographic of anatomical features of PFO in cryptogenic stroke patients compared to autospsy results.

This study retrospectively evaluates the anatomical characteristics of all patients who underwent percutaneous PFO closure for cryptogenic stroke after acceptance by a dedicated HST at two congenital heart disease institutions in the Netherlands (Fig. 1).

## Methods

### Patients

We retrospectively analyzed all consecutive patients who underwent percutaneous PFO closure with a double disc device at Radboudumc (Nijmegen) and Amsterdam UMC (Amsterdam, the Netherlands) between 2016 and 2022. Patients who received PFO closure before the implementation of the HST (before July 2018 for Radboudumc and before January 2016 for Amsterdam UMC) were excluded.

All procedures were performed with either the Amplatzer PFO occluder or the Amplatzer Multifenestrated septal occluder (Abbott, Abbott Park, Illinois, USA). The decision on which device size was most suitable for each patient was left to the discretion of the implanting cardiologist. The decision was based on TEE findings during the procedure.

All patients underwent a standardized work-up to rule out other potential causes of stroke. This included at least 48-hour rhythm monitoring to rule out atrial fibrillation, imaging of the carotid arteries to rule out atherosclerosis, testing for coagulation disorders, and saline contrast echocardiography to assess the RLS. Additional diagnostic tests were performed when indicated. Furthermore, classical cardiovascular risk factors were identified. The Risk of Paradoxical Embolism (RoPE) score was calculated for all patients [15]. Following this assessment, the HST reviewed all cases to determine the potential contribution of the PFO to the stroke. When the patients were assessed, the PFO-Associated Stroke Causal Likelihood (PASCAL) classification was not yet available [10]. This classification may improve patient selection for PFO closure. To assess this in our series, the PASCAL classification was retrospectively applied to the available data for all patients.

### Echocardiography

All patients underwent standard TTE with saline contrast to determine the presence of an RLS and to rule out other cardiac causes of the stroke. Patients with an intracardiac thrombus, severe calcification or stenosis of the aortic or mitral valve were excluded from PFO closure.

All echocardiograms were analyzed by three independent cardiologists (TtC, AD, BB). Classification of the RLS was done by measuring the number of bubbles of agitated saline present in the left atrium within 5 heartbeats after opacification of the right atrium. Patients who did not receive agitated saline as contrast but only color Doppler were excluded. The Valsalva maneuver was applied if there was no shunt at rest or if the referring cardiologist deemed Valsalva necessary to better evaluate the shunt size. Shunt severity was graded in 5 groups: none (no bubbles), small Grade 1 (1–5 bubbles), mild Grade 2 (6–25 bubbles), moderate Grade 3 (> 25 bubbles), and severe Grade 4 (opacification of the left ventricle).

**Fig. 2:**
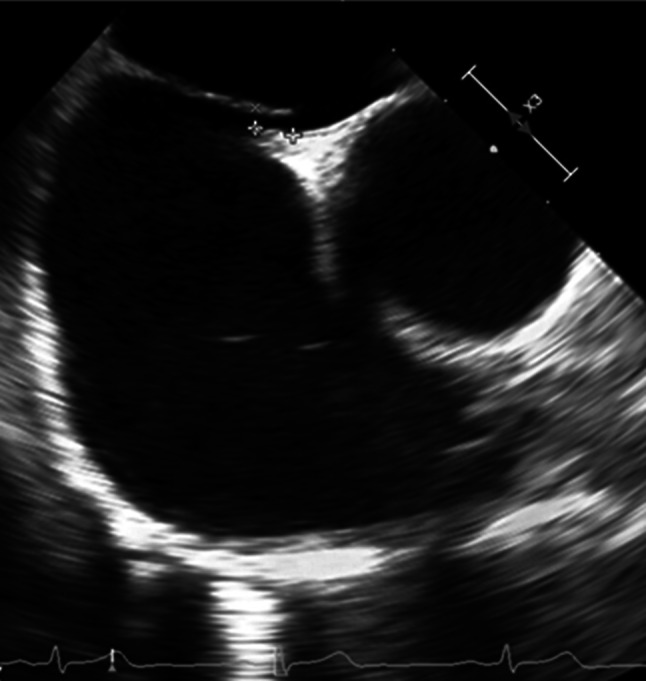
TTE image of an unstretched PFO

**Fig. 3:**
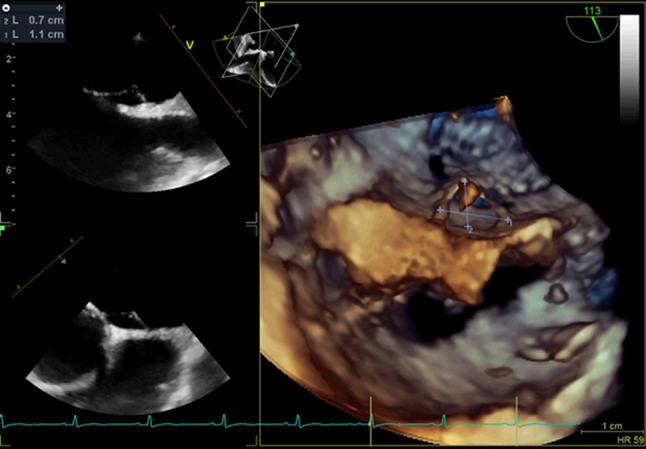
TEE image of a stretched PFO

The results of TEE imaging were also recorded to further assess the PFO characteristics. Anatomical PFO characteristics were measured with standard TEE imaging (Epiq, Philips, Best, the Netherlands, and Vivid E95, GE Healthcare, Horten, Norway). The PFO size was measured with two-dimensional TEE before PFO intervention as an “unstretched diameter” in 114 patients (Fig. 2) or with three-dimensional TEE during PFO intervention, after placement of a guidewire through the PFO, resulting in a maximal “stretched diameter” in 109 patients (Fig. 3). Atrial septal excursion was measured on an image with the best cross section of the atrial septum between 30 to 60 degrees (Fig. 2).

**Fig. 4:**
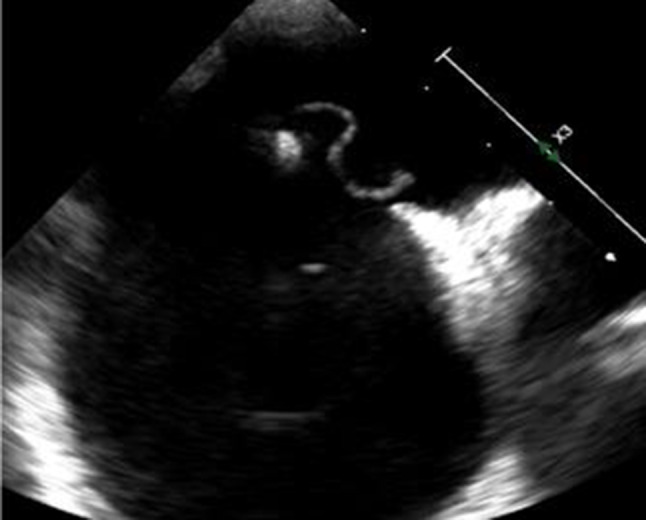
TTE image of a mobile ASA with an excursion of more than 15 mm

As described in the PASCAL score, a risk-enhancing feature was defined as a Grade 3 shunt or higher and/or an ASA with at least 10 mm of excursion from midline (see Fig. 4; [10]).

**Fig. 5:**
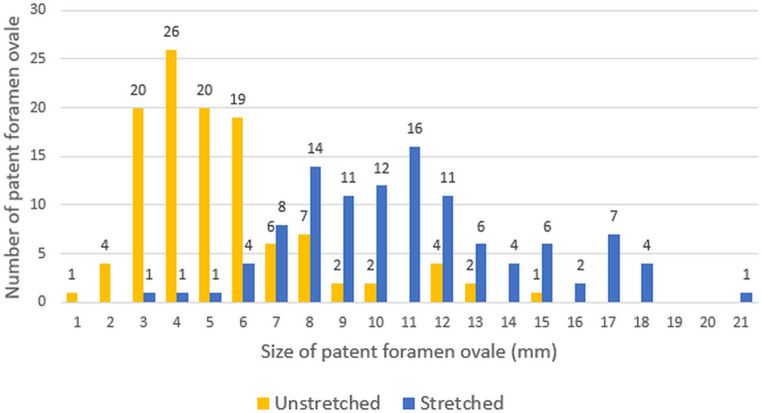
Distribution of PFO sizes between unstretched and stretched measurements. Distribution of the number of patients for each PFO size in mm. In yellow, the unstretched diameter opening, measured using echocardiography. In blue, the stretched diameter opening, measured during the closure procedure using a guidewire

### Statistical analysis

The Shapiro-Wilk Test was performed to test for normality. Categorical variables were summarized as the number of subjects with percentages, continuous variables with normal distribution as mean with standard deviation and continuous variables with non-normal distribution as median with interquartile ranges. Statistical analyses were performed with IBM SPSS Statistics for Windows, version 28 (IBM Corp., Armonk, New York).

## Results

Between January 2016 and April 2022, 223 consecutive patients who underwent percutaneous PFO closure were included, 114 patients at Radboudumc (Nijmegen) and 109 patients at Amsterdam UMC (Amsterdam, the Netherlands). The mean age was 42.8 ± 10.7 years, 115 patients (51.6%) were male, and the median risk of paradoxical embolism (RoPE) score was 7 [IQR 6–8]. The RLS grade at baseline with contrast bubble study was small in 17 patients (7.6%), mild in 43 patients (19.2%), moderate in 67 patients (30.0%), and severe in 96 patients (43.0%). In 44 out of 223 patients (19.7%) Valsalva maneuver was not performed. In total, 73% had a Grade 3 shunt or higher. Of all patients included, 70 (31.4%) had an atrial septal aneurysm (ASA) (≥ 10 mm excursion of the septum). The mean PFO diameter in patients with cryptogenic stroke was 5.3 ± 2.5 mm in the unstretched group and 11.0 ± 3.5 mm in the stretched group (Fig. 5). The PASCAL classification was retrospectively applied to all patients, the classification was unlikely in 14 patients (6.3%), possible in 109 patients (48.9%) and probable in 100 patients (44.8%) (Tab. 1; [10, 15]). Overall, 80% of patients had at least one risk-enhancing feature, as defined in the PASCAL score [10].

**Table 1:** Characteristics of all patients with a PFO

	PFO closure
Number of patients	*n* = 223
Mean age at time of referral, years [SD]	42.8 [± 10.7]
Male, *N* (%)	115 (51.6)
Median RoPE Score, [IQR]	7 [6–8]
PASCAL Classification System, *N* (%)	
*Unlikely*	14 (6.3%)
*Possible*	109 (48.9%)
*Probable*	100 (44.8%)
Shunt grade, *N* (%)	
*Small*	17 (7.6)
*Mild*	43 (19.2)
*Moderate*	67 (30.0)
*Severe*	96 (43.0)
≥ 10 mm excursion of septum, *N* (%)	70 (31.4)
≥ 15 mm excursion of septum, *N* (%)	35 (15.7)
Mean unstretched diameter opening, mm [SD]^*^	5.3 [± 2.5]
Mean stretched diameter opening, mm [SD]^†^	11.0 [± 3.5]
Any residual shunt after 6 months, *N* (%)	64 (28.7)
Any residual shunt after 12 months, *N* (%)	44 (19.7)

Characteristics of patients who underwent PFO closure. Shunt size is measured using contrast bubble study during TTE. The excursion of the septum and unstretched diameter opening are measured using echocardiography. The stretched diameter opening is measured during the closure procedure using a guidewire. Residual shunt is defined as any crossing of bubbles on TTE with contrast bubble study within 5 heartbeats

* *n* = 114 unstretched diameter is measured using TEE, without manipulating the orifice

† *n* = 109 stretched diameter is measured after placing a wire through the PFO orifice during the closure procedure, measuring the maximal stretched diameter

*PASCAL* PFO-associated stroke causal likelihood^14^, *PFO* patent foramen ovale, *RoPE* risk of paradoxical embolis^13^, *TTE* transthoracic echocardiography

In our cohort, 82 patients had a RoPE-score < 7. Of these, 68 patients were classified as having a possible PFO-associated stroke based on the PASCAL classification. This was due to a large shunt in 37 patients, a combination of a large shunt and an ASA in 26 patients, and the presence of an isolated ASA in 5 patients.

The 20% of patients without risk-enhancing features (median RoPE-score of 7, IQR 6–8) were accepted for PFO closure for various reasons: a high RoPE-score (21 patients), absence of an alternative cause of stroke (10 patients), multiple stroke events (7 patients), recurrent event despite antithrombotic therapy (2 patients), presence of Factor V Leiden without an alternative cause (2 patients), antiphospholipid syndrome without an alternative cause (1 patient), and occupation-related risk (1 patient; a flight attendant with a possible increased risk of stroke recurrence).

## Discussion

This study shows that, despite the fact that all PFOs were considered by the HST as contributing factors to the stroke, risk-enhancing anatomical features were not present in 20%. Looking into these risk-enhancing characteristics in more detail, former studies showed that the PFO diameter in patients with a cerebral event was significantly larger (4 ± 2 mm) compared to those without (2 ± 1 mm, *p* < 0.001) [16]. Notably, an unstretched PFO diameter greater than 4 mm was associated with an increased risk of stroke and TIA [16]. In our study, we observed larger unstretched diameters that can be classified as “high-risk”, with a mean of 5.4 ± 2.6 mm compared to the previous report’s mean of 4.0 ± 2.0 mm [16]. This difference may be caused by differences in the methodology used to measure the PFO. Moreover, prior studies demonstrated that the risk of stroke increases with the presence of an ASA. An ASA, defined as an excursion of the interatrial septum > 10 mm, was present in 31.4% of patients in our series. This is in concordance with previous reports [17].

Autopsy studies and cohorts of patients who underwent TEE for reasons other than evaluation of a cardiac origin of stroke reported a prevalence of ASA of about 1–5% [18, 19]. Notably lower than the 32% we observed.

Additionally, the presence of an ASA or a hypermobile septum may facilitate a larger opening of the PFO. Potentially allowing passage of thrombus from the venous circulation through the PFO, formation of thrombus in the tunnel of the PFO or local thrombus formation in the septal aneurysm [20]. Future research is needed to substantiate these hypotheses.

The anatomical characteristics of PFOs associated with stroke are diverse, but a large diameter and the presence of ASA appear to be indicative. Since there is significant heterogeneity in the anatomical features of pathological PFOs, the role these features should play in the decision-making remains unclear. Using the PASCAL score, one risk-enhancing anatomical feature can shift a PFO-related stroke from ‘unlikely’ to ‘possible’. The interpretation of these terms remains difficult. In our study, more patients had a ‘possible’ outcome rather than a ‘probable’ one, and 20% did not have any risk-enhancing features despite all patients having a PFO-associated stroke.

Given the heterogeneity of PFOs and the still high number needed to treat to prevent a single stroke a thorough understanding of the individual patient and a multidisciplinary assessment are essential before deciding on PFO closure [21].

### Limitations

Our study has several limitations. We lack information on the PFO characteristics of patients with a PFO who were declined for percutaneous closure. Therefore, we are not able to make any comparisons. Additionally, while we demonstrate that PFOs can be stretched, resulting in a larger opening, the relationship between this characteristic and stroke risk remains unclear. The stretched diameter can only be obtained with an invasive measurement and is not a useful parameter for identifying patients eligible for PFO closure. Furthermore, due to the lack of information on patients’ baseline characteristics, we are unable to provide a detailed assessment of the selection process or identify potential areas of improvement. Lastly, there may be a larger number of patients with a severe shunt size, as 15% of the patients with a shunt grade lower than severe (opacification) did not undergo a Valsalva maneuver.

## Conclusion and clinical implications

Although all patients accepted for percutaneous PFO closure were individually assessed by a dedicated HST, 20% had a PFO without risk-enhancing features but were still accepted for closure due to other reasons. This highlights the importance of careful individual assessment of young stroke patients with a PFO. Future studies are needed to identify the characteristics that contribute to stroke in these patients, including follow-up for stroke recurrence in patients who were accepted as well as patients who were declined for percutaneous closure.
